# Special Issue “Quantum Foundations: 90 Years of Uncertainty”

**DOI:** 10.3390/e21020159

**Published:** 2019-02-08

**Authors:** Gustavo M. Bosyk, Sebastian Fortin, Pedro W. Lamberti, Federico Holik

**Affiliations:** 1 Instituto de Física La Plata, UNLP, CONICET, Facultad de Ciencias Exactas, 1900 La Plata, Argentina; 2 CONICET, Departamento de Física, Universidad de Buenos Aires, Buenos Aires, C1053 CABA, Argentina; 3 Facultad de Matemática, Astronomía, Física y Computación, Universidad Nacional de Córdoba & CONICET, Córdoba, Argentina

**Keywords:** foundations of quantum mechanics, uncertainty relations, bell inequalities, entropy, quantum computing

The VII Conference on Quantum Foundations: 90 years of uncertainty (https://sites.google.com/site/viijornadasfundamentoscuantica/) was held during November 29th to December 1st, in 2017, at the Facultad de Matemática, Astronomía, Física y Computación, Córdoba, Argentina. It gathered experts in the foundations of quantum mechanics from different countries around the world, interested in promoting a multidisciplinary approach to the fundamental questions of quantum theory and its applications, by taking in consideration not only the physical, but also the philosophical and mathematical aspects of the theory. By those days, 90 years had passed since the seminal paper of Werner Heisenberg [1], describing the reciprocal uncertainty relation between position and momentum in the quantum realm. But the intriguing questions about the interpretation of those relations in connection to the general problems of the interpretation of the quantum formalism, still remain. This was reflected in the vivid discussions that were posed during the Conference.

This special issue captures the main aspects of this debate in connection with other fundamental questions of quantum theory and its applications, by incorporating a selected list of contributions that we now present below.

In the paper “Evaluating the Maximal Violation of the Original Bell Inequality by Two-Qudit States Exhibiting Perfect Correlations/Anticorrelations”, by Andrei Y. Khrennikov and Elena R. Loubenets [2], a general class of symmetric two-qubit states with perfect correlations or anticorrelations between Alice and Bob was introduced. It was proved that, for all states belonging to this class, the maximal violation of the original Bell inequality is upper bounded by a factor $\frac{3}{2}$ and the two-qubit states where this quantum upper bound is attained were given. This is a step forward for solving the problem of finding the quantum upper bound for the original Bell inequality. The experimental implications of these results were also discussed.

In the paper “Revisiting Entanglement within the Bohmian Approach to Quantum Mechanics”, by Claudia Zander and Angel Ricardo Plastino [3], the concept of entanglement was discussed in the framework of the Bohmian approach to quantum mechanics. Using this approach, two partial measures for the amount of entanglement corresponding to a pure state of a pair of quantum particles were constructed. These measures were then put in connection with the notion of total entanglement—that relies on the linear entropy of the single-particle reduced density matrix—which was shown to be equal to their sum. A clear interpretation of the introduced measures was given in terms of the ontology of Bohmian dynamics.

In the paper “New Forms of Quantum Value Indefiniteness Suggest that Incompatible Views on Contexts Are Epistemic”, by Karl Svozil [4], the problem of quantum probabilities and quantum contextuality was addressed. Quantum logics used in extensions of the Kochen–Specker theorem were discussed. The study of these logics and the structure of the probabilistic states that can be built using them, lead the author to suggest a natural interpretation for the quantum formalism. According to this view, quantum systems can be completely characterized by a unique context and a “true” proposition within this context; this situation defines the ontic state of the quantum system. It was argued that, unless there is a total match between preparation and measurement contexts, information about the former from the latter cannot be ontic, but epistemic.

In the paper “Adiabatic Quantum Computation Applied to Deep Learning Networks”, by Jeremy Liu et al. [5], the task of training deep learning networks was addressed. This was done by exploring the possibility of using quantum devices. The authors do this by focusing on a restricted form of adiabatic quantum computation known as quantum annealing, performed by a D-Wave processor. They propose a particular network topology that can be trained to classify MNIST and neutrino detection data. They compared their quantum annealing approach with other extant alternatives, and showed that the quantum approach can find good network parameters in a reasonable time, despite increased network topology complexity.

In the paper “Entropic Uncertainty Relations for Successive Measurements in the Presence of a Minimal Length” by Alexey E. Rastegin [6], the generalized uncertainty principle for successive measurements in the presence of a minimal length was discussed. Uncertainties were described by appealing to generalized entropies of both the Rényi and Tsallis types. The specific features of measurements of observables with continuous spectra were taken into account. It was first shown that, since uncertainty relations formulated in terms of Shannon entropies involve a state-dependent correction term, they will be different, in general, from preparation uncertainty relations. Next, it was shown that state-independent uncertainty relations can be obtained in terms of Rényi and Tsallis entropies. These have the same lower bounds as in the preparation scenario and were shown to depend on the acceptance function of apparatuses in momentum measurements.

In the paper “Quantization and Bifurcation beyond Square-Integrable Wavefunctions”, by Ciann–Dong Yang and Chung–Hsuan Kuo [7], nonsquare-integrable (NSI) solutions of the Schrödinger equation are discussed. These solutions are ruled out in the majority of the formulations of quantum mechanics, due to problems with the conservation of probability. Contrarily, in this paper, a quantum-trajectory approach to energy quantization that includes the possibility of nonsquare- integrable solutions of the Schrödinger equation was considered. It was shown that both, normalized and unnormalized wavefunctions contribute to energy quantization. While square-integrable wavefunctions help to locate the bifurcation points at which energy has a step jump, it turns out that the non square-integrable ones form the flat parts of the stair-like distribution of the quantized energies. The synchronicity between the energy quantization process and the center-saddle bifurcation process was also discussed, in connection to the nonsquare-integrable wave functions.

In the paper “Gudder’s Theorem and the Born Rule”, by Francisco De Zela [8], the Born probability rule was discussed. The author proves that it can be derived from Gudder’s theorem [9]. In doing so, the author tried to identify the fundamental underlying assumptions that lead to a probability rule such as Born’s. It was then argued that Born’s rule applies to both the classical and the quantum domains.

In the paper “Uncertainty Relation Based on Wigner–Yanase–Dyson Skew Information with Quantum Memory” by Jun Li and Shao–Ming Fei [10], uncertainty relations based on Wigner–Yanase– Dyson skew information with quantum memory were studied. The authors derive uncertainty inequalities in product and summation forms. The lower bounds of these inequalities were found and were shown to contain two terms. One of them is related to the degree of compatibility of two measurements. The other one is connected to the quantum correlation between the measured system and the quantum memory.

In the review paper “Uncertainty Relations for Coarse-Grained Measurements: An Overview”, by Fabricio Toscano et al. [11], the problem of uncertainty relations tailored specifically to coarse-grained measurement of continuous quantum observables was addressed, including both theoretical and experimental aspects. These inequalities have applications in detection of quantum correlations and security requirements in quantum cryptography. In order to deal with continuous variable systems, measurements are coarse grained, but the coarse-grained observables do not necessarily obey the same uncertainty relations as the original ones. This leads to the study of coarse-grained uncertainty relations associated to continuous variable quantum systems. This review focused on such uncertainty relations as well as their applications in quantum information theory.

We hope that the selected papers will be of interest for the community of physicists and philosophers working on the foundations of quantum mechanics.
